# A nutrition–inflammation index weighted toward disease burden: the lactate dehydrogenase-to-albumin ratio in *TP53*-mutated myeloid neoplasms

**DOI:** 10.3389/fnut.2026.1966874

**Published:** 2026-10-16

**Authors:** Xiaoqin Xin, Zongqi Wang

**Affiliations:** Department of Clinical Laboratory, Ganzhou Hospital-Nanfang Hospital, Southern Medical University (Ganzhou People's Hospital), Ganzhou, Jiangxi, China

**Keywords:** lactate dehydrogenase-to-albumin ratio, nutrition–inflammation biomarkers, prognosis, prognostic nutritional index, TP53 mutation, acute myeloid leukemia, myelodysplastic syndromes

## Abstract

**Background:**

Albumin-containing composite indices are frequently labeled as nutrition–inflammation biomarkers, although their biological meaning remains uncertain when direct nutritional assessment is unavailable. We investigated the construct validity and interpretive boundaries of the lactate dehydrogenase-to-albumin ratio (LAR) in TP53-mutated acute myeloid leukemia (AML) and myelodysplastic syndromes (MDS). Specifically, we examined whether pretreatment LAR more closely reflected disease burden or systemic inflammatory status, evaluated the relative contributions of its LDH and albumin components, and assessed its outcome associations and incremental prognostic information.

**Methods:**

This retrospective cohort included 116 patients, with 114 adults included in the primary analysis. Associations of continuous log_2_-transformed LAR with prespecified disease-burden, acute-phase, and blood-count-derived markers were evaluated using Spearman correlations with false-discovery-rate control. Cox models assessed overall survival (OS), composite progression-free survival (PFS), and the cause-specific rate of progression or relapse. Sensitivity analyses additionally adjusted for the number of TP53 variants detected, co-occurring myeloid mutations and treatment category. Aalen–Johansen and Fine–Gray methods were used to evaluate progression cumulative incidence while accounting for death before progression as a competing event. Nonlinearity and incremental model performance were also examined.

**Results:**

Among 114 adults, 74 deaths, 93 composite PFS events, 76 progression or relapse events, and 17 deaths before progression occurred. LAR correlated with white blood cell count (ρ = 0.406; q < 0.001) and marrow blast percentage (ρ = 0.298; q = 0.004), but showed no consistent correlation with available acute-phase markers. Component analyses indicated that LDH contributed more strongly than albumin to the outcome-related signal of the ratio. Each doubling of LAR was associated with shorter OS (adjusted hazard ratio [HR], 1.57; 95% confidence interval [CI], 1.20–2.05; *p* < 0.001) and composite PFS (adjusted HR, 1.44; 95% CI, 1.14–1.84; *p* = 0.003). These estimates were essentially unchanged after further adjustment for the number of TP53 variants, co-mutation number and treatment category (OS adjusted HR, 1.64; 95% CI, 1.21–2.21). However, the optimism-corrected increases in C-index were only 0.005 and 0.013, respectively. LAR was associated with the cause-specific rate of progression or relapse (adjusted HR, 1.39; 95% CI, 1.05–1.83; *p* = 0.021), but not with progression cumulative incidence in the pooled cohort after accounting for competing death (subdistribution HR, 1.02; 95% CI, 0.80–1.31; *p* = 0.855); this pooled estimate reflected the AML stratum, and in MDS the subdistribution HR was 1.73 (95% CI, 1.14–2.61). Associations with OS were present within both AML and MDS and were essentially unchanged in a diagnosis-stratified model (OS HR, 1.62; 95% CI, 1.23–2.13); the within-AML estimate for composite PFS was imprecise (HR, 1.33; 95% CI, 0.97–1.81).

**Conclusion:**

In TP53-mutated AML and MDS, higher pretreatment LAR was associated with shorter OS, shorter composite PFS, and a higher cause-specific rate of progression or relapse, but provided only modest incremental discrimination beyond established clinical factors and, in the pooled cohort, was not associated with progression cumulative incidence after accounting for competing death. Its correlations with leukocyte count and marrow blast burden, together with the stronger contribution of LDH than albumin, are more consistent with a disease-burden-weighted signal than with a nutritional one. No validated nutritional assessment and no complete acute-phase marker panel were available, so a nutritional or inflammatory contribution to LAR can be neither confirmed nor excluded. LAR should therefore be interpreted as a disease-burden-weighted prognostic correlate whose nutritional significance requires direct validation, rather than as a standalone nutritional, prognostic, or treatment-selection tool.

## 1 Introduction

TP53-mutated acute myeloid leukemia (AML) and myelodysplastic syndromes (MDS) are associated with adverse outcomes, although prognosis is not uniform. Although one large series of TP53-mutated AML and MDS with excess blasts found no survival differences according to allelic state, clone size, concurrent mutations, or diagnostic category (1), other studies have reported that survival varies according to TP53 allelic state and variant allele frequency (VAF), blast count, complex karyotype, and treatment (2–4). These factors also influence treatment outcomes. Venetoclax plus azacitidine can increase remission rates, but durable survival remains limited in TP53-mutated disease, while transplantation outcomes continue to depend on molecular and cytogenetic risk (5–7). A routinely available laboratory measure may complement these established factors in exploratory prognostic assessment, but its value cannot be inferred from statistical association alone.

The lactate dehydrogenase-to-albumin ratio (LAR) is calculated from two routine laboratory measurements with different and nonspecific determinants. LDH may increase with leukemic cell turnover, tissue injury, hemolysis, hepatic dysfunction, or infection, whereas albumin is influenced by systemic inflammation, hepatic synthetic function, catabolic stress, and nutritional reserve (8–12). Previous studies in AML and MDS have associated LDH or albumin individually with early mortality and survival (9, 13–15). Prognostic associations for LAR have also been reported in diffuse large B-cell lymphoma and in studies conducted predominantly in solid tumors (16, 17). Whether LAR carries similar information in TP53-mutated AML or MDS is unknown, as is whether the ratio marks a recognizable clinical phenotype, whether its association is driven mainly by LDH or by albumin, and whether it adds prognostic information beyond available clinical factors.

Experimental studies have linked mutant p53 and AML with altered glycolysis, marrow lactate accumulation, histone lactylation, and immune remodeling (18, 19). These findings provide a possible biological context but do not establish that circulating LAR measures these processes. Total serum LDH is neither specific to LDHA activity nor a direct measure of lactate production. LAR may instead reflect leukemic burden, systemic inflammation, host condition, or a combination of these features. Correlations with disease-burden and inflammatory markers can describe the associated phenotype but not its mechanism.

Early mortality is common in TP53-mutated disease, which complicates outcome definition. Composite progression-free survival includes both progression or relapse and death. When documented progression is analyzed separately, death prevents its subsequent observation and must be treated as a competing event. Cause-specific and cumulative-incidence models therefore address different questions and may yield different associations (20). In addition, improved model fit does not necessarily indicate a meaningful improvement in patient-level discrimination.

We have previously reported that routine coagulation activation in this registry tracks genomic-instability features but adds little prognostic discrimination beyond cytogenetic and molecular risk (21). Whether an albumin-containing index described as a nutrition–inflammation biomarker behaves in the same way has not been examined. We therefore examined pretreatment LAR in adults with TP53-mutated AML or MDS. LAR was evaluated as a continuous variable in relation to disease-burden and inflammatory markers, overall survival, and composite progression-free survival. The relative contributions of LDH and albumin were examined, and the incremental prognostic information provided by LAR was assessed beyond available clinical covariates. Documented progression was also evaluated using both cause-specific and cumulative-incidence estimands in the presence of competing death. These analyses were exploratory and were intended to define the association and interpretive limits of LAR rather than to derive a clinical cutoff or treatment-selection rule.

## 2 Materials and methods

### 2.1 Study design

This retrospective cohort study was conducted at Ganzhou People’s Hospital. Patients diagnosed with acute myeloid leukemia (AML) or myelodysplastic syndromes (MDS) between 2016 and April 2026 were identified from the institutional clinical and molecular database. Baseline clinical, laboratory, cytogenetic, and molecular data were abstracted from institutional records. Follow-up information was obtained from subsequent institutional medical records and, when available, telephone follow-up, which was used to update vital status and selected major treatment events occurring at outside institutions. The data cutoff for outcome ascertainment was June 2026.

Patients were eligible if they had a confirmed diagnosis of AML or MDS, a TP53 mutation detected by next generation sequencing, complete pretreatment serum lactate dehydrogenase and albumin measurements, receipt of clinical management according to institutional treatment standards, and more than 1 month of clinical follow-up. A total of 116 patients met all eligibility criteria. The primary analysis was restricted to the 114 adults aged 18 years or older. The remaining two patients were aged 13 and 9 years and were included only in the sensitivity analysis of the full cohort. No formal sample size calculation was performed because all eligible patients identified during the predefined study period were included. This registry has also supported a published analysis of pretreatment coagulation parameters in TP53-mutated AML and MDS (21). Eligibility differed between the two studies, so the cohorts overlap rather than nest; the exposure, endpoints and analyses are distinct.

The index date for all time-to-event analyses was the date of the diagnostic bone-marrow examination at which TP53-mutated AML or MDS was established. Overall survival, composite progression-free survival, time to progression and all cumulative-incidence estimates are therefore measured in months from diagnosis.

Serum LDH and albumin formed part of the routine diagnostic panel and were obtained during the same admission as the diagnostic bone-marrow examination, in every case before the first dose of disease-directed therapy; patients whose only available LDH or albumin result post-dated the start of therapy were not eligible, no post-treatment value was carried back, and no repeat measurement was substituted for the baseline value. Because the laboratory, marrow, cytogenetic and molecular assessments all belonged to a single diagnostic episode, the interval between LAR measurement and the index date was shorter than the one-month resolution at which follow-up time was recorded, and no patient contributed unexposed person-time between the index date and measurement of the exposure. Eligibility nevertheless required more than 1 month of clinical follow-up, so patients who died within the first month after diagnosis could not be included and the earliest observed death occurred in month 2. This is a selection criterion rather than an immortal-time interval.

The study was conducted in accordance with the Declaration of Helsinki and was approved by the Ethics Committee of Ganzhou People’s Hospital (approval no. PJB2025-145-03). The requirement for informed consent was waived because of the retrospective observational design and analysis of de-identified clinical and follow-up data.

### 2.2 Molecular and treatment covariates

TP53 variant status was abstracted from the diagnostic next-generation sequencing report and was classified solely according to the number of distinct TP53 variants documented: two or more TP53 variants, a single TP53 variant, or indeterminate variant number. The indeterminate category comprised only the affected TP53 exon without variant-level detail, so that the number of variants could not be established retrospectively. No other criterion was applied. In particular, 17p copy number, del(17p) by fluorescence *in situ* hybridization and single-nucleotide-polymorphism array testing for copy-neutral loss of heterozygosity were not systematically available, and variant allele frequency was not used to infer loss of the wild-type allele. This variable is therefore a variant-count surrogate and is not equivalent to the consensus multi-hit or biallelic definition used in the WHO and International Consensus classifications; it is reported as the number of TP53 variants throughout. Co-occurring myeloid gene mutations were recorded from the same targeted panel and were summarized both as the number of non-TP53 driver mutations per patient and as individual gene frequencies.

Treatment was classified as chemotherapy or hypomethylating-agent-based therapy, supportive care only, or allogeneic hematopoietic stem-cell transplantation (allo-HSCT). Transplantation status was obtained from institutional records or telephone follow-up received at outside institutions. All patients were analyzed from the index TP53-mutated AML or MDS diagnosis. Lines of therapy delivered after documented relapse or progression were not abstracted and were therefore not modeled, and no analysis was restricted to, or conditioned on, relapsed or refractory disease.

### 2.3 LAR and inflammatory variables

LAR was recalculated as LDH (U/L)/albumin (g/L) and modeled as log_2_(LAR), making effect estimates interpretable per doubling. Neutrophil-to-lymphocyte ratio (NLR) = ANC/ALC; platelet-to-lymphocyte ratio (PLR) = platelets/ALC; lymphocyte-to-monocyte ratio (LMR) = ALC/AMC; systemic immune-inflammation index (SII) = platelets×ANC/ALC; absolute monocytes (AMC) = WBC × monocyte percentage/100; systemic inflammation response index (SIRI) = ANC × AMC/ALC; aggregate index of systemic inflammation (AISI) = platelets×ANC × AMC/ALC; prognostic nutritional index (PNI) = albumin+5 × ALC; CRP-to-albumin ratio (CAR) = CRP/albumin; fibrinogen-to-albumin ratio (FAR) = fibrinogen/albumin; and albumin-to-globulin ratio (AGR) = albumin/globulin. CAR, FAR, PNI, and AGR share albumin with LAR and were designated mathematically coupled secondary variables. IL-2, IL-4, IL-6, IL-10, TNF-α, and IFN-γ were available for only 5–6 patients each and were excluded from formal inference without imputation.

### 2.4 Outcome definitions and competing events

Primary survival endpoints included overall survival (OS), composite progression-free survival (composite PFS), and time to disease-specific progression. OS was defined as the time from the index date defined above to death from any cause. Vital status and, when applicable, date of death were ascertained from institutional medical records and supplemented by telephone follow-up. Patients alive were censored at the most recent date on which vital status could be established. Composite PFS was defined as the time from the index date to the first occurrence of either disease progression or all-cause death; patients without either event were censored at the last evaluable follow-up. Time to progression was defined as the time from the index date until documented disease progression; deaths prior to progression were treated as a competing risk event in Fine–Gray competing-risk regression. Event times were available only as integer months, and nine patients had documented progression or relapse and death recorded in the same month. In the primary analysis these ties were resolved in favor of progression: the patient contributed a progression or relapse event in that month and the subsequent death did not contribute to the competing-event count, because progression had been explicitly documented in the clinical record before death. No artificial time offset was applied and no fractional month was imputed. The opposite rule, under which all nine records were classified as competing deaths, was applied as a prespecified sensitivity analysis.

Progression and relapse were ascertained by the treating hematologist and were re-adjudicated for this analysis against the source bone-marrow, blood-count and cytogenetic records, using published criteria appropriate to each diagnosis.

For AML, the European LeukemiaNet 2022 recommendations were applied (22). In patients who had achieved complete remission, relapsed disease was defined as at least 5% bone-marrow blasts, reappearance of circulating blasts in at least two peripheral-blood samples obtained at least 1 week apart, or development of new extramedullary disease. Patients who had not achieved complete remission, complete remission with incomplete hematological recovery or complete remission with partial hematological recovery by the applicable response landmark—after two cycles of intensive chemotherapy, or approximately 180 days after commencing less-intensive therapy - were classified as having refractory disease, and the date of that landmark assessment was taken as the progression date. Patients diagnosed before 2022 were re-classified retrospectively against the same criteria so that a single definition applied across the whole study period.

For MDS, the modified International Working Group 2006 response criteria were applied (23). Relative to the pretreatment marrow blast percentage, progression required an increase in bone-marrow blasts of at least 50% to more than 5% (baseline below 5%), to more than 10% (baseline 5–10%), to more than 20% (baseline 10–20%) or to more than 30% (baseline 20–30%); in the absence of a rise in blasts, progression could also be recorded as a decrement of at least 50% from the maximum response in granulocytes or platelets, a fall in hemoglobin of at least 2 g/dL, or new transfusion dependence. Transformation to AML was recorded when bone-marrow or peripheral-blood blasts reached 20% or more, consistent with the WHO and International Consensus classifications, and accounted for most progression events in the MDS group. Every change had to be confirmed on a repeat assessment and to be independent of a reversible cause such as infection, bleeding or treatment-related cytopenia.

Because the AML and MDS progression definitions are not interchangeable, progression analyses are reported separately by diagnosis as well as for the pooled cohort, and the pooled estimates are interpreted alongside the diagnosis-specific ones.

### 2.5 Statistical analysis

Continuous variables are summarized as median (IQR) and categorical variables as n (%). Median follow-up was estimated by the reverse Kaplan–Meier method. Associations of log_2_ LAR with biological and inflammatory variables used Spearman rank correlation with 1,000 bootstrap resamples for 95% CIs. The Benjamini–Hochberg procedure controlled the false-discovery rate across a prespecified family of 16 markers. Four of these (PNI, CAR, FAR and AGR) share albumin with LAR; they were retained for transparency but were not interpreted as independent corroboration. The AML–MDS comparison was exploratory and is reported with a nominal *p* value.

Cox proportional-hazards models estimated associations with OS, composite PFS, and the cause-specific hazard of progression/relapse. Covariates were selected on clinical grounds and data availability rather than univariable significance. Association models were unadjusted; clinically adjusted for age, sex, TP53 VAF, AML/MDS, and complex karyotype; and burden-adjusted by additionally including log_2_ WBC and marrow blast percentage. A component model entered log_2_ LDH and albumin per 5-g/L increase simultaneously with the clinical covariates. Restricted cubic splines with knots at the 5th, 35th, 65th, and 95th percentiles assessed departure from log-linearity. Median LAR was used only for descriptive visualization and was not treated as an optimized threshold.

To explore incremental prognostic information, a base clinical model containing age, sex, TP53 VAF, AML/MDS, and complex karyotype was compared with the nested model after adding continuous log_2_ LAR, separately for OS and composite PFS. Model fit was compared with a likelihood-ratio test, and discrimination was summarized by Harrell’s C-index (24, 25). Internal validation used 500 bootstrap resamples: both models were refitted in each resample, their C-indices were evaluated in the resample and in the original cohort, and mean optimism was subtracted from apparent performance (24). The reported optimism-corrected ΔC-index is the difference between the corrected C-indices. No feature selection, threshold derivation, calibration analysis, or decision-curve analysis was performed; these comparisons were prespecified as exploratory and do not constitute development of a clinical prediction model.

The proportional-hazards assumption was evaluated using Grambsch–Therneau score tests based on scaled Schoenfeld residuals and rank-transformed event time, both for LAR and globally across all covariates in each Cox model. Covariate-specific tests were also examined, with a two-sided *p* < 0.05 considered evidence of potential nonproportionality. If a violation had been detected, the prespecified strategy was to incorporate time-varying effects or apply stratified Cox models when clinically appropriate.

Aalen–Johansen estimators described cumulative incidence, and Fine–Gray models quantified subdistribution hazards for progression/relapse and death before documented progression/relapse (20). Fine–Gray coefficients were interpreted as associations with cumulative incidence, not instantaneous cause-specific rates. Progression models used the clinical and burden covariate sets. Because only 17 deaths occurred before documented progression/relapse, the corresponding models were limited to unadjusted and age-adjusted estimates; full clinical adjustment was an exploratory sensitivity analysis. Inflammation-marker prognostic analyses used separate clinically adjusted Cox models reported per IQR, with FDR correction across markers. Sensitivity analyses included both pediatric records, adult LAR winsorized at the 1st and 99th percentiles, and reclassification of the nine same-month outcomes. The clinically adjusted models were further extended by adding the number of TP53 variants, treatment intensity (supportive care only versus active disease-directed therapy) and the number of co-occurring myeloid mutations, entered separately and simultaneously, and were re-estimated after excluding allo-HSCT recipients, after excluding patients managed with supportive care only, after restricting the cohort to patients with a determinate TP53 variant count, and after recoding single-variant patients with a TP53 variant allele frequency above 50% as probably biallelic. The bootstrap-corrected incremental C-index analysis was repeated with these molecular and treatment variables added to the base model.

Two further sets of analyses addressed marker missingness and diagnostic heterogeneity. First, because C-reactive protein and ferritin were measured at the discretion of the treating team rather than by protocol, patients with and without each measurement were compared on the prespecified clinical and laboratory characteristics using Mann–Whitney and Fisher exact tests, and measurement status was entered as a covariate in clinically adjusted Cox models for OS and composite PFS to test whether it was associated with outcome. Second, because AML and MDS differ in natural history, baseline hazard, treatment and progression definition, the clinically adjusted LAR models were refitted separately within each diagnosis, refitted as Cox models stratified by diagnosis (allowing a separate baseline hazard for AML and MDS), and tested for a LAR-by-diagnosis interaction using a likelihood-ratio test; the interaction test is reported as exploratory because the study was not powered for it. A small number of *post hoc* correlations between log_2_ LAR and routinely measured laboratory variables with a partial relationship to hepatic synthetic function and nutritional reserve (cholinesterase, total cholesterol, triglycerides) are reported without multiplicity adjustment and are explicitly hypothesis-generating; they are not a substitute for direct nutritional assessment.

All tests were two-sided and were conducted in accordance with the REMARK recommendations (26, 27). Analyses were conducted in Python 3.12 (lifelines v0.28.0, scikit-survival v0.22.2, statsmodels v0.14.2, pandas v2.2, NumPy v1.26, SciPy v1.13). Competing-risks cumulative incidence was estimated with the Aalen–Johansen estimator implemented in lifelines.

## 3 Results

### 3.1 Cohort characteristics and clinical outcomes

The primary cohort comprised 114 adults: 60 (52.6%) with MDS and 54 (47.4%) with AML. Median age was 65 years (IQR, 59–71); 61 patients (53.5%) were male and 82 (71.9%) had a complex karyotype. Median TP53 VAF was 49.8% (IQR, 25.1–78.3), median marrow blasts were 14.6% (IQR, 4.8–44.0), and median LAR was 7.01 (IQR, 5.21–12.18). Two or more TP53 variants were documented in 56 patients (49.1%), a single TP53 variant in 37 (32.5%), and the variant number was indeterminate in 21 (18.4%). Co-occurring myeloid gene mutations were detected in 76 patients (66.7%; median 1, IQR 0–2), most frequently DNMT3A (15; 13.2%), TET2 (14; 12.3%), FLT3-ITD (9; 7.9%), NPM1 (8; 7.0%) and ASXL1 (8; 7.0%). Ninety-one patients (79.8%) received chemotherapy or hypomethylating-agent-based therapy, 18 (15.8%) received supportive care only and 5 (4.4%) underwent allo-HSCT (Table 1). Reverse Kaplan–Meier median follow-up was 22 months.

**Table 1:** Clinical and laboratory characteristics of the adult primary cohort.

Characteristic	Adult cohort (*n* = 114)
Age, years	65.0 (59.0–71.0)
Male sex	61 (53.5%)
MDS	60 (52.6%)
AML	54 (47.4%)
Complex karyotype	82 (71.9%)
TP53 VAF, %	49.8 (25.1–78.3)
TP53 variants documented, ≥2	56 (49.1%)
TP53 variants documented, 1	37 (32.5%)
TP53 variant number indeterminate	21 (18.4%)
≥1 co-occurring myeloid mutation	76 (66.7%)
Co-occurring mutations per patient, n	1 (0–2)
Marrow blasts, %	14.6 (4.8–44.0)
WBC, ×10^9^/L	3.2 (2.0–7.9)
LDH, U/L	281.6 (200.1–425.0)
Albumin, g/L	37.8 (33.5–41.6)
LAR, U/g	7.01 (5.21–12.18)
CRP, mg/L	32.4 (15.2–97.9); n = 56
Ferritin, ng/mL	540.7 (330.0–1019.1); n = 84
Fibrinogen, g/L	3.73 (2.44–4.88)
Documented progression/relapse	76 (66.7%)
Death before progression/relapse	17 (14.9%)
Deaths from any cause	74 (64.9%)
Chemotherapy or hypomethylating agent	91 (79.8%)
Supportive care only	18 (15.8%)
Allogeneic HSCT during follow-up	5 (4.4%)

Values are median (IQR) or n (%). CRP was available in 56 and ferritin in 84 adults. TP53 status was classified only by the number of distinct variants documented and is not equivalent to the consensus multi-hit definition; 17p copy number and copy-neutral loss of heterozygosity were not assessed, and TP53 variant number was indeterminate in 21 patients (exon-level report only). Co-occurring mutations are non-TP53 myeloid driver mutations detected on the same targeted panel, most frequently DNMT3A (*n* = 15), TET2 (*n* = 14), FLT3-ITD (*n* = 9), NPM1 (*n* = 8) and ASXL1 (*n* = 8). Treatment categories are mutually exclusive and describe therapy recorded after the index diagnosis. LAR, LDH-to-albumin ratio; VAF, variant allele frequency; HSCT, hematopoietic stem-cell transplantation.

Seventy-four deaths and 76 documented progression/relapse events occurred. Seventeen patients died before any documented progression/relapse; accordingly, composite PFS included 93 events, whereas the competing-risk endpoint comprised 76 progression/relapse events, 17 competing deaths, and 21 censored observations. Median OS was 12 months and median composite PFS was 5 months.

### 3.2 LAR correlates with disease burden and selected inflammatory indices

Higher log_2_ LAR correlated with WBC (ρ = 0.406; 95% bootstrap CI, 0.220–0.562; FDR q < 0.001), marrow blasts (ρ = 0.298; 95% CI, 0.116–0.474; q = 0.004), and, more weakly, TP53 VAF (ρ = 0.217; 95% CI, 0.022–0.403; q = 0.047) (Table 2; Figures 1a–c). In an exploratory diagnostic-group comparison, median LAR was higher in AML than in MDS (8.97 vs. 6.39; nominal *p* = 0.004); this comparison was not used to define a threshold.

**Table 2:** Prespecified phenotype correlations of continuous log_2_ LAR.

Marker	*n*	Spearman ρ	95% bootstrap CI	*P*	FDR q
WBC	114	0.406	0.220 to 0.562	<0.001	<0.001
Marrow blasts	114	0.298	0.116 to 0.474	0.001	0.004
TP53 VAF	114	0.217	0.022 to 0.403	0.020	0.047
NLR	104	0.135	−0.055 to 0.327	0.173	0.251
PLR	104	−0.405	−0.560 to −0.206	<0.001	<0.001
LMR	103	−0.447	−0.595 to −0.273	<0.001	<0.001
SII	104	−0.085	−0.280 to 0.118	0.389	0.414
SIRI	104	0.429	0.259 to 0.577	<0.001	<0.001
AISI	104	0.309	0.135 to 0.475	0.001	0.004
CRP	56	0.195	−0.079 to 0.447	0.149	0.238
Ferritin	84	0.115	−0.101 to 0.332	0.299	0.342
Fibrinogen	114	0.021	−0.175 to 0.209	0.825	0.825
PNI*	104	0.121	−0.087 to 0.315	0.220	0.271
CAR*	56	0.213	−0.062 to 0.439	0.114	0.203
FAR*	114	0.119	−0.076 to 0.309	0.209	0.271
AGR*	114	−0.206	−0.383 to −0.017	0.028	0.056

Bootstrap CIs used 1,000 resamples. Benjamini–Hochberg q values were calculated across the full prespecified 16-marker family shown here. Figure 1a displays the 12 non-mathematically coupled markers. *PNI, CAR, FAR, and AGR share albumin with LAR and should not be interpreted as independent biological corroboration.

**Figure 1:**
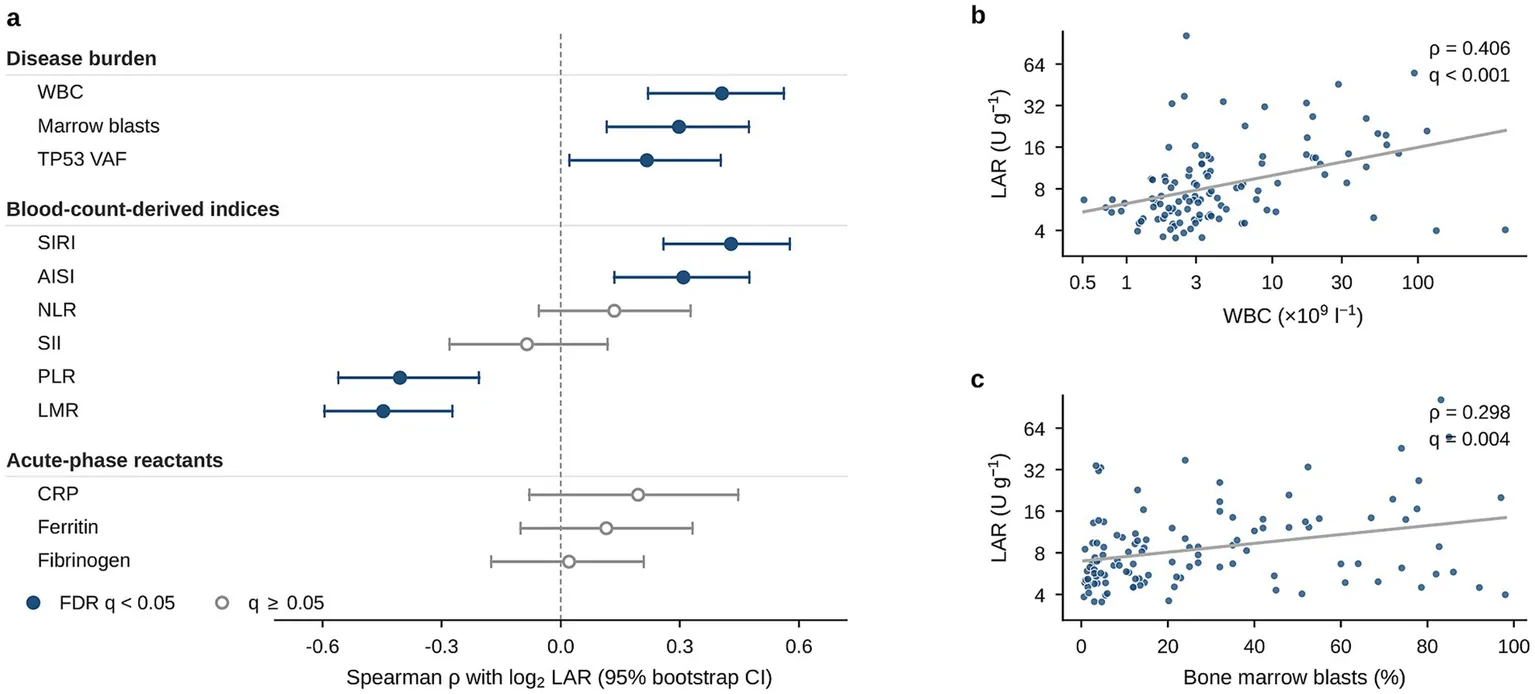
Disease-burden and inflammatory correlates of LAR. **(a)** Spearman correlations of continuous log_2_ LAR with the prespecified marker family, grouped by marker type. Points and bars show Spearman ρ and 95% bootstrap CIs; filled points indicate FDR q < 0.05 across the full 16-marker family in Table 2 and open points q ≥ 0.05. The four albumin-coupled indices (PNI, CAR, FAR and AGR) are retained in Table 2 but omitted here because they share albumin with LAR. **(b)** LAR versus white blood cell count. **(c)** LAR versus bone marrow blast percentage. Both axes are logarithmic where the distribution is right-skewed; fitted lines are visual summaries and inference uses Spearman correlation, with coefficients quoted to three decimal places as in Table 2. LAR, LDH-to-albumin ratio; WBC, white blood cell count; VAF, variant allele frequency.

Among non-coupled inflammatory markers, log_2_ LAR correlated positively with SIRI (ρ = 0.429; q < 0.001) and AISI (ρ = 0.309; q = 0.004), and inversely with PLR (ρ = −0.405; q < 0.001) and LMR (ρ = −0.447; q < 0.001). NLR, SII, CRP, ferritin and fibrinogen showed no FDR-significant correlation, although CRP and ferritin were measured in only 56 (49.1%) and 84 (73.7%) patients respectively, so these null results characterize the acute-phase markers that were available rather than excluding an inflammatory contribution (Table 2; Figure 1a). The pattern therefore followed leukocyte and blast burden and some count-derived indices, without a uniform acute-phase response. PNI, CAR, FAR, and AGR were retained in Table 2 but were not interpreted as orthogonal evidence because each shares albumin with LAR.

### 3.3 Continuous LAR is associated with OS and composite PFS

Each doubling of LAR was associated with higher mortality in the clinically adjusted model (HR, 1.57; 95% CI, 1.20–2.05; *p* < 0.001) and burden-adjusted model (HR, 1.63; 95% CI, 1.22–2.19; *p* < 0.001). Corresponding estimates for composite PFS were 1.44 (95% CI, 1.14–1.84; *p* = 0.003) and 1.48 (95% CI, 1.15–1.90; *p* = 0.002) (Table 3). Restricted cubic splines showed no evidence of nonlinearity for OS (*p* = 0.766) or composite PFS (*p* = 0.596), supporting continuous log-linear modeling over the observed range (Figures 2a,b).

**Table 3:** Core continuous LAR and component associations across clinical estimands.

Endpoint	Exposure	Clinical model	*P*	n/events	Burden-adjusted model	*P*
		HR/sHR (95% CI)			HR/sHR (95% CI)	
Primary analysis: continuous LAR						
OS	Continuous LAR	HR 1.57 (1.20–2.05)	<0.001	114/74	HR 1.63 (1.22–2.19)	<0.001
Composite PFS	Continuous LAR	HR 1.44 (1.14–1.84)	0.003	114/93	HR 1.48 (1.15–1.90)	0.002
Progression (cause-specific)	Continuous LAR	HR 1.39 (1.05–1.83)	0.021	114/76	HR 1.43 (1.07–1.92)	0.017
Progression (Fine–Gray)	Continuous LAR	sHR 1.02 (0.80–1.31)	0.855	114/76	sHR 1.10 (0.83–1.46)	0.500
Component-specific analysis						
OS	LDH per doubling*	HR 1.53 (1.16–2.03)	0.003	114/74	–	–
OS	Albumin per 5 g/L*	HR 0.86 (0.67–1.10)	0.222	114/74	–	–
Composite PFS	LDH per doubling*	HR 1.38 (1.08–1.78)	0.011	114/93	–	–
Composite PFS	Albumin per 5 g/L*	HR 0.83 (0.67–1.02)	0.072	114/93	–	–

LAR effects are per doubling. Clinical models adjust for age, sex, TP53 VAF, AML/MDS, and complex karyotype; burden models additionally include log_2_ WBC and marrow blasts. Cause-specific HRs describe the progression/relapse rate among patients still event-free; Fine–Gray sHRs describe relative cumulative incidence with pre-progression death competing. *Component models include log_2_ LDH and albumin simultaneously with the clinical covariates. OS, overall survival; PFS, progression-free survival; HR, hazard ratio; sHR, subdistribution hazard ratio.

**Figure 2:**
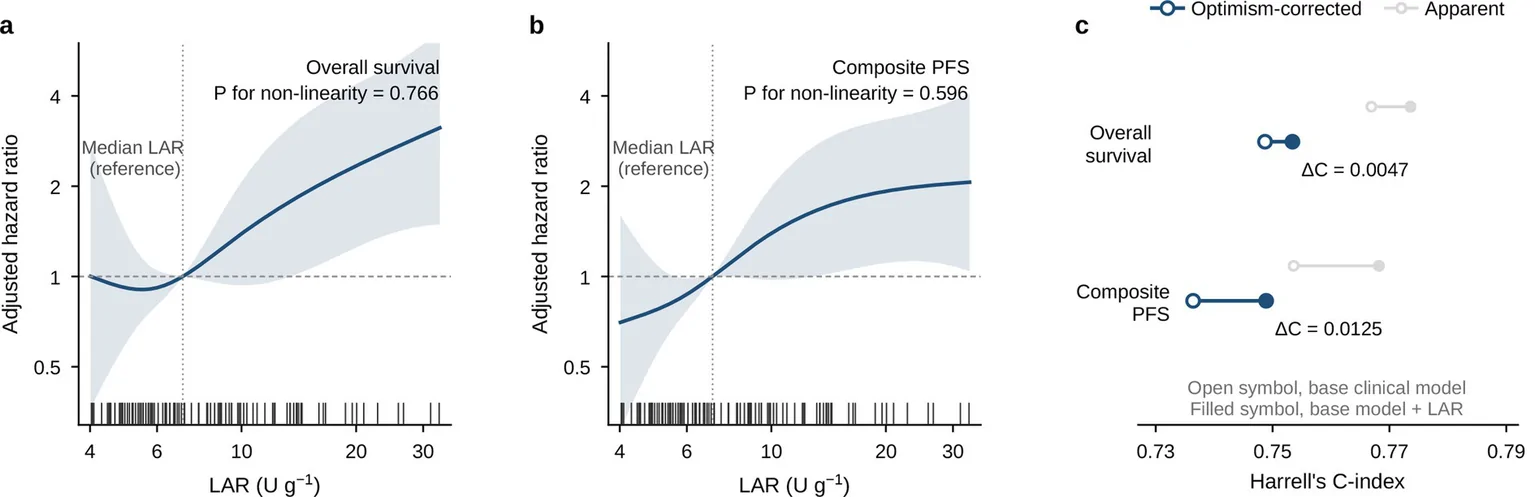
Functional form and incremental prognostic information for continuous LAR. **(a,b)** Clinically adjusted restricted cubic-spline relationships between LAR and **(a)** overall survival and **(b)** composite progression-free survival, centered at the median LAR. Curves span the 5th to 95th percentiles, shading denotes 95% CIs, and rug marks show the observed distribution. **(c)** Exploratory bootstrap internal validation of the base clinical model with and without LAR. Harrell C-indices were corrected for mean optimism across 500 resamples; each segment joins the base clinical model (open symbol) to the same model with LAR added (filled symbol), so that the length of each dark segment is the optimism-corrected change in C-index. The apparent, uncorrected values for the same two models are plotted above each pair in grey, and the longer grey segments show the optimism that the correction removes. LAR, LDH-to-albumin ratio; PFS, progression-free survival.

In the exploratory nested-model comparison, adding continuous LAR improved model fit for OS (likelihood-ratio x^2^ = 10.063; *p* = 0.002) and composite PFS (x^2^ = 8.415; *p* = 0.004). After 500-resample bootstrap optimism correction, the OS C-index was 0.7487 for the base clinical model and 0.7534 after adding LAR (ΔC = 0.0047); corresponding PFS values were 0.7364 and 0.7489 (ΔC = 0.0125). All 500 resamples were successfully fitted. LAR therefore improved model fit but added little discrimination in the derivation cohort (Figure 2c; Supplementary Table S1).

Schoenfeld-residual diagnostics did not identify evidence against proportional hazards. Across unadjusted, clinical, and burden-adjusted models for OS, composite PFS, and cause-specific progression/relapse, global *p* values ranged from 0.393 to 0.687 and LAR-specific *p* values from 0.393 to 0.881 (Supplementary Table S2).

In simultaneous component models, LDH per doubling remained associated with OS (HR, 1.53; 95% CI, 1.16–2.03; *p* = 0.003) and composite PFS (HR, 1.38; 95% CI, 1.08–1.78; *p* = 0.011). Albumin per 5-g/L increment was not associated with OS (HR, 0.86; 95% CI, 0.67–1.10; *p* = 0.222) and had an imprecise inverse association with composite PFS (HR, 0.83; 95% CI, 0.67–1.02; *p* = 0.072) (Table 3). These mutually adjusted component estimates suggest that the ratio signal was weighted more strongly toward LDH, but they are not a mediation analysis.

### 3.4 Competing-risk analysis separates event rate from cumulative incidence

For documented progression/relapse, the cause-specific association persisted in the clinical model (HR per LAR doubling, 1.39; 95% CI, 1.05–1.83; *p* = 0.021) and burden-adjusted model (HR, 1.43; 95% CI, 1.07–1.92; *p* = 0.017). In contrast, the pooled Fine–Gray associations with progression/relapse cumulative incidence showed no evidence of an effect, with confidence intervals that remained wide: clinically adjusted sHR, 1.02 (95% CI, 0.80–1.31; *p* = 0.855) and burden-adjusted sHR, 1.10 (95% CI, 0.83–1.46; *p* = 0.500) (Table 3; Figures 3a,b). In the pooled cohort, LAR was therefore associated with the progression rate among patients still alive and progression-free, but not with the overall probability of observing progression in the presence of competing death; the diagnosis-specific estimates below qualify this.

**Figure 3:**
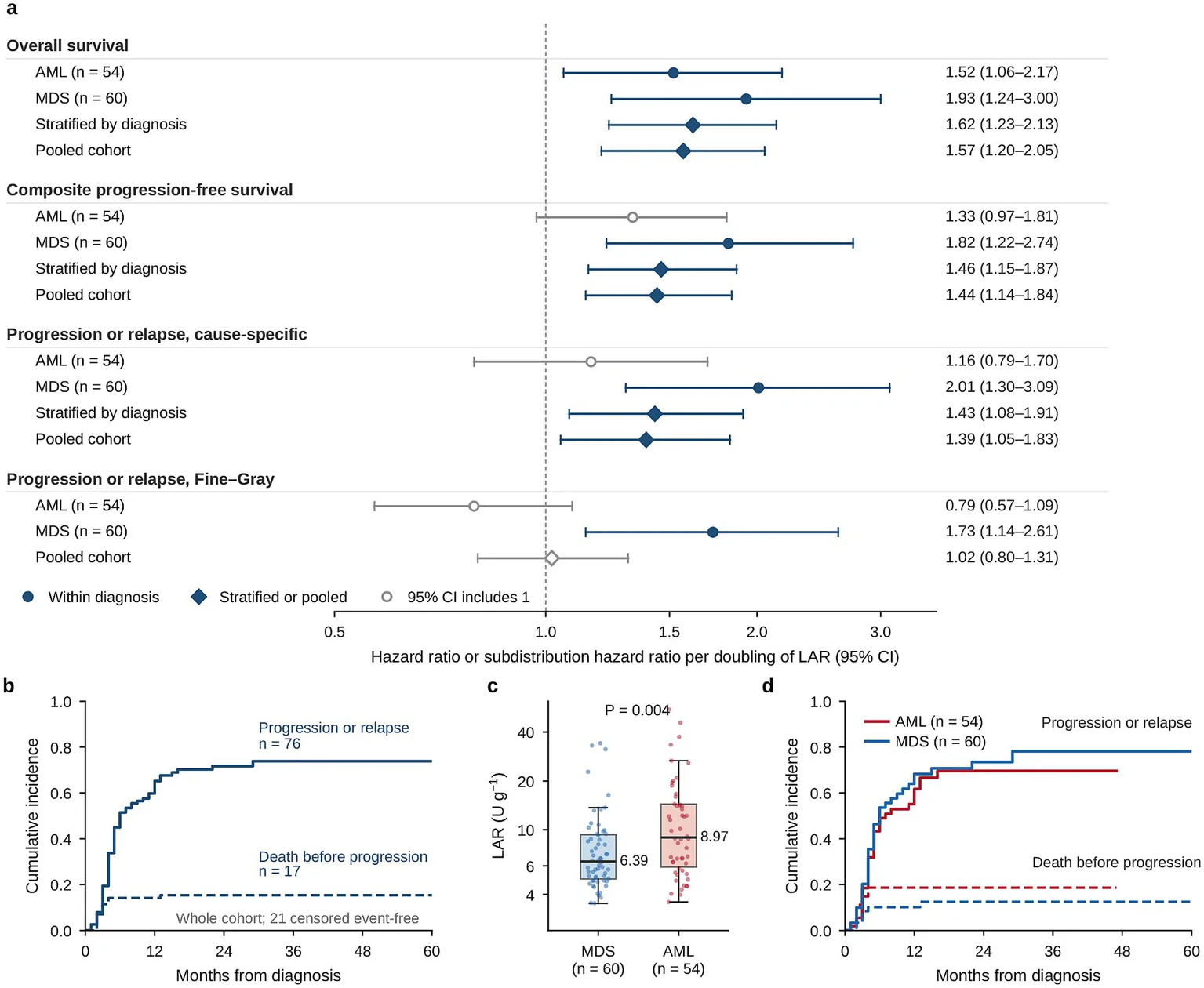
Outcome associations of LAR by estimand and diagnosis. **(a)** Adjusted HRs or subdistribution HRs per doubling of LAR for overall survival, composite progression-free survival, and progression or relapse in AML, MDS, diagnosis-stratified models, and the pooled cohort. Circles indicate diagnosis-specific estimates, diamonds stratified or pooled estimates, and open symbols indicate 95% CIs including 1. Models were adjusted for age, sex, TP53 VAF and complex karyotype; pooled models additionally included diagnosis. No diagnosis-stratified Fine–Gray model was fitted. **(b)** Aalen–Johansen cumulative incidence of progression or relapse and death before progression in the whole cohort (76 and 17 events, respectively; 21 patients censored event-free). **(c)** Distribution of LAR by diagnosis (median 8.97 in AML vs. 6.39 in MDS; nominal *p* = 0.004). **(d)** Diagnosis-specific cumulative incidence of progression or relapse and death before progression. AML, acute myeloid leukemia; MDS, myelodysplastic syndromes; LAR, LDH-to-albumin ratio; HR, hazard ratio.

For death before documented progression/relapse, the unadjusted sHR was 1.79 (95% CI, 1.18–2.73; *p* = 0.006) and the parsimonious age-adjusted sHR was 1.90 (95% CI, 1.23–2.94; *p* = 0.004). The exploratory full clinical estimate attenuated and crossed the null (sHR, 1.55; 95% CI, 0.95–2.54; *p* = 0.080). Because only 17 such events occurred, these estimates were treated as secondary and are reported in Supplementary Table S3 rather than the main tables.

### 3.5 Inflammatory comparators and sensitivity analyses

In separate clinically adjusted models standardized per IQR, LAR remained associated with both OS (HR, 1.74; FDR q = 0.013) and composite PFS (HR, 1.57; FDR q = 0.037). NLR, PLR, LMR, SII, SIRI, AISI, CRP, ferritin, fibrinogen, PNI, CAR, FAR, and AGR did not retain FDR-significant prognostic associations. Ferritin showed only a trend for OS (HR, 1.47; nominal *p* = 0.064; FDR q = 0.450) (Supplementary Table S4). The cytokine panel was too sparse for formal inference.

Including all 116 patients yielded similar clinical estimates for OS (HR, 1.57; 95% CI, 1.23–2.01; *p* < 0.001) and composite PFS (HR, 1.46; 95% CI, 1.17–1.83; *p* < 0.001); adult LAR winsorization also produced concordant estimates (Supplementary Table S5). Reclassifying all nine same-month progression/death records as competing deaths left the clinically adjusted progression sHR null (0.95; 95% CI, 0.71–1.26; *p* = 0.712) (Supplementary Table S3).

LAR did not track the molecular or treatment characteristics of the cohort. Median LAR was 7.23 (IQR, 5.38–12.12) in patients with two or more TP53 variants, 8.50 (6.21–13.93) in single-variant patients and 5.84 (4.65–10.99) when the variant number was indeterminate (Kruskal–Wallis *p* = 0.165; two or more versus single variant *p* = 0.477). Median LAR did not differ across treatment categories (*p* = 0.299) or between patients with and without a co-occurring mutation (7.08 versus 6.90; *p* = 0.812), and log_2_ LAR was not correlated with the number of co-mutations (ρ = −0.118; *p* = 0.212).

Adding the number of TP53 variants, treatment intensity or co-mutation number to the clinical model left the association of LAR with OS essentially unchanged (HR per doubling 1.70, 1.52 and 1.53, respectively), as did adding all three simultaneously (HR, 1.64; 95% CI, 1.21–2.21; *p* = 0.001); corresponding composite PFS estimates were 1.58, 1.43, 1.46 and 1.58 (fully adjusted 95% CI, 1.21–2.04; *p* = 0.001). The divergence between estimands also persisted: the fully adjusted cause-specific HR for progression or relapse was 1.61 (95% CI, 1.19–2.19; *p* = 0.002), whereas the corresponding Fine–Gray subdistribution HR remained close to the null (1.07; 95% CI, 0.83–1.38; *p* = 0.611) (Supplementary Table S6). Estimates were similarly stable after excluding the five allo-HSCT recipients (OS HR, 1.46; 95% CI, 1.11–1.93), after excluding the 18 patients managed with supportive care only (OS HR, 1.64; 95% CI, 1.23–2.20), after restricting the analysis to the 93 patients with a determinate TP53 variant count (OS HR, 1.57; 95% CI, 1.13–2.17) and after recoding single-variant patients with a variant allele frequency above 50% as probably biallelic (OS HR, 1.49; 95% CI, 1.10–2.02) (Supplementary Table S7). When the number of TP53 variants, treatment intensity and co-mutation number were added to the base model, the optimism-corrected increase in the C-index after adding LAR remained small (0.012 for OS and 0.018 for composite PFS). In clinically adjusted models that did not include LAR, supportive care only was associated with shorter OS (HR, 2.38; 95% CI, 1.26–4.51; *p* = 0.008), whereas co-mutation number (OS HR, 0.87; 95% CI, 0.71–1.05) and, among the 93 patients with a determinate variant count, two or more versus a single TP53 variant (OS HR, 1.05; 95% CI, 0.61–1.82) were not.

### 3.6 Marker missingness and disease-subgroup analyses

Acute-phase markers were measured at clinical discretion, and measurement was not random. Patients in whom CRP was measured had a higher TP53 VAF (median 64.0% versus 43.5%; *p* = 0.024) and lower albumin (34.7 versus 39.8 g/L; *p* = 0.002) than those in whom it was not, consistent with testing being prompted by suspected infection or clinical deterioration. Patients in whom ferritin was measured had a lower TP53 VAF (42.8% versus 73.4%; *p* < 0.001), higher albumin (38.1 versus 33.9 g/L; *p* = 0.025) and lower LAR (6.6 versus 9.9; *p* = 0.029). Measurement status was not associated with outcome after clinical adjustment, either for CRP (OS HR, 1.15; 95% CI, 0.72–1.85; *p* = 0.554) or for ferritin (OS HR, 0.85; 95% CI, 0.49–1.48; *p* = 0.561), and the groups did not differ in age, sex, diagnosis, karyotype, treatment category or TP53 variant count (Supplementary Table S8). The null correlations between LAR and CRP or ferritin should therefore be read as an absence of a consistent association within a non-randomly measured subset, not as evidence against an inflammatory component of LAR.

The association between LAR and outcome was present within both diagnostic groups and was not an artifact of pooling AML with MDS. In AML (*n* = 54), each doubling of LAR was associated with shorter OS (HR, 1.52; 95% CI, 1.06–2.17; *p* = 0.022) and, less precisely, with composite PFS (HR, 1.33; 95% CI, 0.97–1.81; *p* = 0.072). In MDS (*n* = 60) the estimates were larger (OS HR, 1.93; 95% CI, 1.24–3.00; *p* = 0.004; composite PFS HR, 1.82; 95% CI, 1.22–2.74; *p* = 0.004), despite MDS having the lower median LAR of the two groups (6.39 versus 8.97) (Figure 3c). A Cox model stratified by diagnosis, which allows a separate baseline hazard for AML and MDS, gave estimates close to the pooled analysis (OS HR, 1.62; 95% CI, 1.23–2.13; *p* < 0.001; composite PFS HR, 1.46; 95% CI, 1.15–1.87; *p* = 0.002). The exploratory LAR-by-diagnosis interaction was not significant for either endpoint (likelihood-ratio *p* = 0.465 for OS and *p* = 0.393 for composite PFS), although the study was not powered to detect one (Figure 3a; Supplementary Table S9).

The competing-risk pattern, in contrast, did differ by diagnosis. In MDS, higher LAR was associated with documented progression on both estimands (cause-specific HR, 2.01; 95% CI, 1.30–3.09; *p* = 0.002; Fine–Gray sHR, 1.73; 95% CI, 1.14–2.61; *p* = 0.009), whereas in AML neither estimate reached significance (cause-specific HR, 1.16; 95% CI, 0.79–1.70; Fine–Gray sHR, 0.79; 95% CI, 0.57–1.09) (Figures 3a,d). The null pooled Fine–Gray estimate therefore reflects the AML stratum, in which death competes more strongly with documented progression, rather than a uniform absence of association across the cohort.

In *post hoc*, multiplicity-unadjusted analyses of routine laboratory variables with a partial relationship to hepatic synthetic function and nutritional reserve, log_2_ LAR correlated inversely with cholinesterase (ρ = −0.287; *n* = 48; *p* = 0.048) and positively with triglycerides (ρ = 0.286; *n* = 59; *p* = 0.028), and was not correlated with total cholesterol (ρ = −0.146; *n* = 59; *p* = 0.271) or hemoglobin (ρ = −0.136; *n* = 114; *p* = 0.148). These variables are not validated measures of nutritional status, the analyses were exploratory, and the sample sizes were small; they are reported for transparency and indicate that a nutritional or hepatic synthetic contribution to LAR cannot be excluded by the present data.

## 4 Discussion

In this retrospective cohort of adults with TP53-mutated AML or MDS, pretreatment LAR was associated with both disease phenotype and outcome, although its added predictive value was limited. Higher LAR correlated with WBC, marrow blast percentage, and, more weakly, TP53 VAF, whereas associations with acute-phase markers were inconsistent. Each doubling of LAR was associated with shorter OS and composite PFS after adjustment for clinical characteristics and measures of disease burden, with no evidence of departure from log-linearity. Higher LAR was also associated with the cause-specific rate of documented progression or relapse, but not, in the pooled cohort, with its cumulative incidence when death before progression was treated as a competing event. Adding LAR improved model fit, although the optimism-corrected increases in the C-index were only 0.005 for OS and 0.013 for composite PFS. LAR is therefore a marker of outcome weighted towards disease burden, and not a validated prediction tool.

Previous work has generally examined LDH, albumin, or LAR in broader hematologic or solid-tumor populations. In Chinese Hakka AML cohorts, higher LDH and lower albumin were associated with 60-day mortality; serial LDH carried prognostic information in MDS, and low albumin was associated with poorer outcomes in azacitidine-treated MDS (9, 13–15). The two LAR studies cited above were conducted in diffuse large B-cell lymphoma and, for the most part, in solid tumors (16, 17). AML studies have also reported prognostic associations for NLR, SII, and the CRP-to-albumin ratio (28–30). Our findings are directionally consistent with the adverse associations reported for LDH, albumin, and LAR, but direct comparison is limited by differences in disease definition, treatment exposure, biomarker cutoffs, and outcome selection. In particular, short-term mortality in broader AML cohorts and survival in a TP53-mutated AML or MDS cohort represent different clinical settings. The lack of FDR-significant associations for the inflammatory comparators in our cohort may reflect its molecularly defined case mix, limited sample size, missingness for some markers, and stricter control for multiple testing.

The associations of continuous LAR with OS and composite PFS were similar after inclusion of the two pediatric patients and after winsorization, and spline analyses provided no support for a threshold effect. Adjustment for WBC and marrow blast percentage did not materially attenuate the estimates. However, simultaneous component models showed that LDH retained associations with OS and composite PFS, whereas albumin did not. The adverse progression association was also confined to the cause-specific model, and the gain in discrimination after LAR was added to the clinical model was small. The survival association was therefore reproducible across several model specifications, but the ratio did not demonstrate a distinct biological signal or a clinically important improvement in patient-level risk classification.

These patterns suggest that much of the LAR signal may arise from LDH and its relationship to leukemic burden. LDH may increase with rapid cell proliferation and turnover, but it is also affected by tissue injury, hemolysis, infection, and hepatic dysfunction (8). Correlations with WBC and marrow blasts, together with the component models, support a burden-related interpretation. Albumin may contribute information related to inflammation, hepatic synthetic function, capillary leakage, catabolic stress, or nutritional reserve (10–12), although its mutually adjusted association was weak and imprecise. The correlations with SIRI, AISI, PLR, and LMR may reflect leukemic myeloid expansion, lymphopenia, and thrombocytopenia as well as inflammation. The absence of FDR-significant correlations with NLR, SII, CRP, ferritin and fibrinogen does not support describing LAR as a general inflammatory index, but neither does it exclude an inflammatory component. CRP and ferritin were measured in 49.1 and 73.7% of patients at clinical discretion, and the measured and unmeasured groups differed systematically in TP53 VAF and albumin, so these markers are missing not at random. What these data support is narrower: LAR showed no consistent association with the acute-phase markers that were available in this cohort. Studies of mutant p53-driven glycolysis, AML metabolic reprogramming, marrow lactate, histone lactylation, and immune remodeling provide a plausible metabolic context (18, 19, 31–33), but circulating total LDH is not specific to LDHA activity and does not directly measure lactate production or metabolic flux. The present data therefore cannot establish that the clinical association reflects glycolysis, lactate accumulation, or lactylation.

This cohort contains no validated nutritional assessment, and body-mass index, body-weight trajectory, recent weight loss, dietary intake, NRS-2002, PG-SGA, GLIM criteria and muscle mass were not recorded. The data can therefore show that LAR tracks leukocyte and blast burden, and that the outcome signal is weighted towards LDH rather than albumin, but they cannot show that nutritional status does not contribute to LAR. The exploratory inverse correlation with cholinesterase is consistent with some contribution from hepatic synthetic function or nutritional reserve, although it is unadjusted for multiplicity and based on 48 patients. The claim supported by these data is therefore an operational one: LAR should not be used as a nutritional index in the absence of direct nutritional validation. Whether nutritional status contributes to LAR in TP53-mutated myeloid neoplasms remains open and requires a study in which nutrition is measured directly.

Analyzing AML and MDS separately did not weaken the main result. LAR was associated with OS and composite PFS within each diagnosis, the estimates were if anything larger in MDS despite its lower median LAR, and a diagnosis-stratified model reproduced the pooled estimates; the overall association is therefore not an artifact of AML contributing both higher LAR and worse survival. The competing-risk analyses did differ by diagnosis. In MDS, where progression is largely transformation to AML and where death before transformation is less frequent, LAR was associated with progression on both the cause-specific and the subdistribution scale. In AML, where early death competes strongly with documented relapse, neither estimate reached significance. The null pooled subdistribution hazard therefore comes from the AML stratum and from the event structure, not from a uniform absence of association, and it shows how the choice of estimand and of population governs what a competing-risk analysis can detect. These subgroup estimates are nonetheless based on 54 and 60 patients, the interaction tests were not significant and the study was not powered for them, so the diagnosis-specific results should be read as descriptive.

The cause-specific model estimates the progression or relapse rate among patients who remain alive and progression free, whereas the Fine–Gray model estimates relative cumulative incidence after accounting for the fact that death prevents subsequent progression from being observed (34). Patients with higher LAR may progress more rapidly while still at risk and also be more likely to die before progression is documented. The association between LAR and death before progression is compatible with this explanation, although it was based on only 17 events and should be interpreted cautiously. The two estimates therefore diverge because of the event structure, not because the models disagree. A significant likelihood ratio test indicates improved model fit, but the small optimism-corrected changes in the C-index show that LAR contributed little to patient ranking. Association, model fit, and clinically useful prediction should therefore not be treated as equivalent.

This study has several limitations. Its retrospective design, single-center setting, modest sample size, and lack of an external validation cohort limit generalizability and leave scope for selection bias and residual confounding. Eligibility required complete LDH and albumin data and more than 1 month of follow-up, which may have reduced representation of untreated patients and those with very early death. AML and MDS were combined to obtain an analyzable molecularly defined cohort, but diagnostic heterogeneity remains, and the study was underpowered for reliable subgroup interactions. The number of TP53 variants, co-occurring mutations and treatment category were available and did not materially alter the LAR estimates, but important gaps remain. TP53 status was classified only by variant count, which is not equivalent to the consensus multi-hit definition: 17p copy number, del(17p) and copy-neutral loss of heterozygosity were not assessed, so patients with a single TP53 variant plus 17p loss would have been misclassified as single-variant disease. Consistent with this, 21 of the 37 single-variant patients (56.8%) had a variant allele frequency above 50% and 28 (75.7%) had a complex karyotype, and recoding the former as probably biallelic left the LAR estimates essentially unchanged. Variant number was also indeterminate in 21 patients whose earlier-period reports gave only exon-level annotation. This misclassification is likely to be non-differential with respect to LAR and would bias any comparison between TP53 categories towards the null, which may explain why variant count was not itself prognostic here. Detailed treatment information and exact treatment dates were incomplete, with some outside-institution allo-HSCT identified through telephone follow-up. Vital status was supplemented by telephone follow-up in some patients, whereas progression or relapse required clinical documentation and may therefore have been under-ascertained after transfer of care. The observed associations cannot therefore be considered treatment independent or causal. Event times were recorded in integer months, which left the order of nine same-month progression and death records ambiguous; a sensitivity analysis using the opposite tie-breaking rule gave concordant results. Only 17 deaths occurred before documented progression or relapse. Missing CRP and ferritin values and sparse cytokine measurements also limited the evaluation of systemic inflammation, and because these markers were measured at clinical discretion the missingness is informative rather than random. No validated nutritional assessment of any kind was available, so no conclusion about the nutritional determinants of LAR can be drawn from these data. Subgroup analyses by diagnosis were descriptive and the interaction tests were underpowered. Finally, bootstrap correction provides internal rather than external validation, and calibration, reclassification, decision-curve analysis, and clinical utility were not assessed.

The study also has methodological strengths. The analysis was confined to a molecularly defined TP53-mutated cohort rather than a broad AML or MDS population. LAR was modeled continuously, and splines were used to assess its functional form, avoiding a sample-specific cutoff. Phenotype analyses used a prespecified marker family with FDR control and distinguished markers that were mathematically coupled through albumin. Component models examined the relative contributions of LDH and albumin. The outcome analysis separated the cause-specific rate of progression from its cumulative incidence under competing mortality, while incremental information was assessed using both model fit and bootstrap-corrected discrimination. None of these steps establishes clinical utility, which would require external validation.

Overall, pretreatment LAR appears to be an accessible but nonspecific marker of an adverse, disease-burden-weighted phenotype in TP53-mutated AML or MDS. Its associations with OS and composite PFS were consistent, but its relation to documented progression depended on the estimand, and its incremental discrimination was modest. Prospective multicenter studies should validate continuous LAR within current WHO and ICC classifications, incorporate copy-number-resolved TP53 allelic classification and complete co-mutation profiles, record treatment and event dates precisely, and evaluate calibration and decision-analytic value. Correlative studies that include LDH isoenzymes, serum and marrow lactate, acute-phase proteins, and direct measures of blast metabolism and the marrow microenvironment could clarify the source of the signal. Until such evidence is available, LAR should be regarded as a hypothesis-generating prognostic correlate rather than a treatment-selection marker, a standalone prediction tool, or a surrogate for inflammation or altered lactate metabolism.

## 5 Conclusion

In TP53-mutated AML and MDS, higher pretreatment LAR was associated with adverse outcomes but added little discrimination beyond established clinical variables. The correlations with leukocyte and blast burden fit a disease-burden-weighted signal better than a nutritional one, but no validated nutritional assessment was available and a nutritional contribution cannot be excluded. LAR should not be used as a standalone nutritional assessment or treatment-selection tool without direct nutritional validation.

## Data Availability

The datasets presented in this article are not readily available because they contain patient-level clinical data and are subject to ethical restrictions. Requests to access the datasets should be directed to the corresponding author.
