# Supplementary material for: A nutrition–inflammation index weighted toward disease burden: the lactate dehydrogenase-to-albumin ratio in TP53-mutated myeloid neoplasms
Source: Front Nutr. 2026 Oct 16;13:1966874. doi: 10.3389/fnut.2026.1966874 (PMC13719407; doi:10.3389/fnut.2026.1966874)
Supplement: Supplementary file 1 [file Table_1.docx]

# Supplementary Tables

**Supplementary Table S1. Exploratory bootstrap internal validation of incremental prognostic information.**

| **Outcome** | **Apparent C, base** | **Apparent C, +LAR** | **Corrected C, base** | **Corrected C, +LAR** | **Corrected ΔC** | **LRT χ² (P)** |
| --- | --- | --- | --- | --- | --- | --- |
| OS | 0.7669 | 0.7736 | 0.7487 | 0.7534 | 0.0047 | 10.063 (0.002) |
| Composite PFS | 0.7536 | 0.7682 | 0.7364 | 0.7489 | 0.0125 | 8.415 (0.004) |

The base clinical model included age, sex, *TP53* VAF, AML/MDS, and complex karyotype; +LAR added continuous log_2_ LAR. Harrell's C-indices were corrected for mean optimism across 500 bootstrap resamples; all 500 fits succeeded. Likelihood-ratio tests (LRTs) compared the nested models. These derivation-cohort analyses are exploratory and do not establish calibration, external validity, or clinical utility.

**Supplementary Table S2. Model-level and LAR-specific proportional-hazards diagnostics.**

| **Outcome/model** | **n/events** | **Global χ² (df)** | **Global P** | **LAR χ²** | **LAR P** |
| --- | --- | --- | --- | --- | --- |
| OS; unadjusted | 114/74 | 0.59 (1) | 0.442 | 0.59 | 0.442 |
| OS; clinical | 114/74 | 5.04 (6) | 0.539 | 0.53 | 0.466 |
| OS; burden-adjusted | 114/74 | 6.76 (8) | 0.563 | 0.11 | 0.739 |
| Composite PFS; unadjusted | 114/93 | 0.16 (1) | 0.687 | 0.16 | 0.687 |
| Composite PFS; clinical | 114/93 | 5.16 (6) | 0.524 | 0.02 | 0.881 |
| Composite PFS; burden-adjusted | 114/93 | 5.67 (8) | 0.684 | 0.43 | 0.511 |
| Progression; unadjusted | 114/76 | 0.73 (1) | 0.393 | 0.73 | 0.393 |
| Progression; clinical | 114/76 | 5.42 (6) | 0.492 | 0.39 | 0.533 |
| Progression; burden-adjusted | 114/76 | 6.92 (8) | 0.545 | 0.66 | 0.417 |

Grambsch–Therneau score tests used Schoenfeld residuals and rank-transformed event time. P<0.05 was considered evidence of potential nonproportionality; no global or LAR-specific test met this criterion. Clinical and burden covariates are defined in Table 3.

**Supplementary Table S3. Secondary competing-risk analyses.**

| **Outcome** | **Model** | **Event classification** | **Reclassified, n** | **Events** | **sHR per doubling** | **95% CI** | **P** |
| --- | --- | --- | --- | --- | --- | --- | --- |
| Pre-progression death | Unadjusted Fine–Gray | Primary | 0 | 17 | sHR 1.79 | 1.18–2.73 | 0.006 |
| Pre-progression death | Age-adjusted Fine–Gray | Primary | 0 | 17 | sHR 1.90 | 1.23–2.94 | 0.004 |
| Pre-progression death | Clinical Fine–Gray sensitivity | Primary | 0 | 17 | sHR 1.55 | 0.95–2.54 | 0.080 |
| Progression/relapse | Clinical Fine–Gray | Same-month as deaths | 9 | 67 | sHR 0.95 | 0.71–1.26 | 0.712 |
| Pre-progression death | Age-adjusted Fine–Gray | Same-month as deaths | 9 | 26 | sHR 1.95 | 1.37–2.79 | <0.001 |

Primary classification treated the nine same-month progression/death records as progression because progression was explicitly documented; sensitivity classification treated all nine as competing deaths. The full clinical pre-progression-death model is exploratory because only 17 primary events occurred. Clinical covariates were age, sex, *TP53* VAF, AML/MDS, and complex karyotype.

**Supplementary Table S4. Clinically adjusted prognostic associations of LAR and inflammatory comparators.**

| **Marker** | **Outcome** | **n** | **Events** | **IQR on model scale** | **Adjusted HR/IQR** | **95% CI** | **P** | **FDR q** |
| --- | --- | --- | --- | --- | --- | --- | --- | --- |
| LAR | OS | 114 | 74 | 1.225 | 1.74 | 1.25–2.41 | <0.001 | 0.013 |
| LAR | PFS | 114 | 93 | 1.225 | 1.57 | 1.17–2.10 | 0.003 | 0.037 |
| NLR | OS | 104 | 67 | 1.946 | 0.96 | 0.68–1.36 | 0.839 | 0.901 |
| NLR | PFS | 104 | 85 | 1.946 | 0.98 | 0.74–1.31 | 0.913 | 0.913 |
| PLR | OS | 104 | 67 | 2.424 | 0.89 | 0.65–1.21 | 0.448 | 0.832 |
| PLR | PFS | 104 | 85 | 2.424 | 0.88 | 0.67–1.16 | 0.364 | 0.687 |
| LMR | OS | 103 | 66 | 2.545 | 0.80 | 0.57–1.12 | 0.198 | 0.832 |
| LMR | PFS | 103 | 84 | 2.545 | 0.90 | 0.66–1.21 | 0.479 | 0.687 |
| SII | OS | 104 | 67 | 2.677 | 0.91 | 0.68–1.22 | 0.535 | 0.832 |
| SII | PFS | 104 | 85 | 2.677 | 0.93 | 0.72–1.20 | 0.573 | 0.687 |
| SIRI | OS | 103 | 66 | 4.096 | 1.12 | 0.83–1.51 | 0.456 | 0.832 |
| SIRI | PFS | 103 | 84 | 4.096 | 1.08 | 0.81–1.44 | 0.589 | 0.687 |
| AISI | OS | 103 | 66 | 4.559 | 1.07 | 0.80–1.43 | 0.651 | 0.901 |
| AISI | PFS | 103 | 84 | 4.559 | 1.03 | 0.78–1.36 | 0.835 | 0.899 |
| CRP | OS | 56 | 39 | 2.690 | 1.17 | 0.71–1.92 | 0.532 | 0.832 |
| CRP | PFS | 56 | 45 | 2.690 | 1.22 | 0.79–1.88 | 0.362 | 0.687 |
| Ferritin | OS | 84 | 54 | 1.627 | 1.47 | 0.98–2.20 | 0.064 | 0.450 |
| Ferritin | PFS | 84 | 69 | 1.627 | 1.12 | 0.78–1.60 | 0.537 | 0.687 |
| Fibrinogen | OS | 114 | 74 | 0.999 | 0.94 | 0.63–1.39 | 0.745 | 0.901 |
| Fibrinogen | PFS | 114 | 93 | 0.999 | 1.11 | 0.79–1.57 | 0.550 | 0.687 |
| PNI* | OS | 104 | 67 | 10.023 | 0.95 | 0.82–1.10 | 0.493 | 0.832 |
| PNI* | PFS | 104 | 85 | 10.023 | 1.03 | 0.95–1.11 | 0.505 | 0.687 |
| CAR* | OS | 56 | 39 | 2.961 | 1.21 | 0.73–2.02 | 0.460 | 0.832 |
| CAR* | PFS | 56 | 45 | 2.961 | 1.27 | 0.81–1.98 | 0.290 | 0.687 |
| FAR* | OS | 114 | 74 | 1.156 | 1.02 | 0.69–1.51 | 0.901 | 0.901 |
| FAR* | PFS | 114 | 93 | 1.156 | 1.20 | 0.85–1.68 | 0.301 | 0.687 |
| AGR* | OS | 114 | 74 | 0.445 | 0.97 | 0.72–1.30 | 0.830 | 0.901 |
| AGR* | PFS | 114 | 93 | 0.445 | 0.93 | 0.71–1.22 | 0.584 | 0.687 |

Each marker was entered in a separate model adjusted for age, sex, *TP53* VAF, AML/MDS, and complex karyotype. Right-skewed markers were log_2_-transformed, so one patient with a monocyte count of zero contributes to the Spearman correlations for SIRI and AISI in Table 2 (n=104) but not to the corresponding models here (n=103). No missing values were imputed. Benjamini–Hochberg q values were calculated across the 14 markers within each outcome. *PNI, CAR, FAR and AGR share albumin with LAR and are not independent of it.

**Supplementary Table S5. Sensitivity analyses for continuous LAR.**

| **Population/variant** | **Outcome** | **n** | **Events** | **HR per doubling** | **95% CI** | **P** |
| --- | --- | --- | --- | --- | --- | --- |
| All 116 patients | OS | 116 | 76 | 1.57 | 1.23–2.01 | <0.001 |
| All 116 patients | PFS | 116 | 95 | 1.46 | 1.17–1.83 | <0.001 |
| Adults; LAR winsorized 1st-99th | OS | 114 | 74 | 1.58 | 1.20–2.08 | 0.001 |
| Adults; LAR winsorized 1st-99th | PFS | 114 | 93 | 1.45 | 1.13–1.86 | 0.003 |

Models adjust for age, sex, *TP53* VAF, AML/MDS, and complex karyotype. Adult winsorization was applied to LAR at the 1st and 99th percentiles.

**Supplementary Table S6. Continuous LAR after extended adjustment for *TP53* variant number, treatment intensity and co-occurring mutations.**

| **Endpoint** | **Model** | **n/events** | **Estimate per LAR doubling (95% CI)** | **P** |
| --- | --- | --- | --- | --- |
| OS | Clinical model (primary analysis) | 114/74 | HR 1.57 (1.20–2.05) | <0.001 |
| OS | + number of *TP53* variants | 114/74 | HR 1.70 (1.27–2.27) | <0.001 |
| OS | + treatment intensity | 114/74 | HR 1.52 (1.16–2.00) | 0.002 |
| OS | + number of co-occurring mutations | 114/74 | HR 1.53 (1.16–2.01) | 0.003 |
| OS | + all three simultaneously | 114/74 | HR 1.64 (1.21–2.21) | 0.001 |
| Composite PFS | Clinical model (primary analysis) | 114/93 | HR 1.44 (1.14–1.84) | 0.003 |
| Composite PFS | + number of *TP53* variants | 114/93 | HR 1.58 (1.22–2.04) | <0.001 |
| Composite PFS | + treatment intensity | 114/93 | HR 1.43 (1.12–1.82) | 0.004 |
| Composite PFS | + number of co-occurring mutations | 114/93 | HR 1.46 (1.15–1.87) | 0.002 |
| Composite PFS | + all three simultaneously | 114/93 | HR 1.58 (1.21–2.04) | 0.001 |
| Progression (cause-specific) | Clinical model (primary analysis) | 114/76 | HR 1.39 (1.05–1.83) | 0.021 |
| Progression (cause-specific) | + number of *TP53* variants | 114/76 | HR 1.62 (1.20–2.19) | 0.002 |
| Progression (cause-specific) | + treatment intensity | 114/76 | HR 1.38 (1.04–1.82) | 0.027 |
| Progression (cause-specific) | + number of co-occurring mutations | 114/76 | HR 1.40 (1.05–1.86) | 0.020 |
| Progression (cause-specific) | + all three simultaneously | 114/76 | HR 1.61 (1.19–2.19) | 0.002 |
| Progression (Fine–Gray) | Clinical model (primary analysis) | 114/76 | sHR 1.02 (0.80–1.31) | 0.855 |
| Progression (Fine–Gray) | + number of *TP53* variants | 114/76 | sHR 1.07 (0.83–1.38) | 0.581 |
| Progression (Fine–Gray) | + treatment intensity | 114/76 | sHR 1.03 (0.80–1.31) | 0.835 |
| Progression (Fine–Gray) | + number of co-occurring mutations | 114/76 | sHR 1.02 (0.79–1.30) | 0.894 |
| Progression (Fine–Gray) | + all three simultaneously | 114/76 | sHR 1.07 (0.83–1.38) | 0.611 |

All models include the primary clinical covariate set (age, sex, *TP53* VAF, AML/MDS and complex karyotype) plus continuous log_2_ LAR. Number of *TP53* variants was entered as two indicator variables (two or more variants, and indeterminate variant number, with a single documented variant as reference); treatment intensity as supportive care only versus active disease-directed therapy; and co-occurring mutations as the count of non-*TP53* myeloid driver mutations. Cause-specific HRs describe the progression or relapse rate among patients still event-free; Fine–Gray sHRs describe relative cumulative incidence with pre-progression death competing. HR, hazard ratio; sHR, subdistribution hazard ratio.

**Supplementary Table S7. Treatment- and *TP53*-classification sensitivity analyses for continuous LAR.**

| **Analysis** | **Outcome** | **n** | **Events** | **HR per LAR doubling (95% CI)** | **P** |
| --- | --- | --- | --- | --- | --- |
| Excluding allogeneic HSCT recipients | OS | 109 | 73 | 1.46 (1.11–1.93) | 0.007 |
| Excluding allogeneic HSCT recipients | Composite PFS | 109 | 91 | 1.40 (1.09–1.79) | 0.008 |
| Excluding supportive care only | OS | 96 | 60 | 1.64 (1.23–2.20) | 0.001 |
| Excluding supportive care only | Composite PFS | 96 | 77 | 1.54 (1.19–1.99) | 0.001 |
| Chemotherapy or hypomethylating agent only | OS | 91 | 59 | 1.49 (1.09–2.02) | 0.011 |
| Chemotherapy or hypomethylating agent only | Composite PFS | 91 | 75 | 1.48 (1.13–1.93) | 0.004 |
| Determinate *TP53* variant count only | OS | 93 | 60 | 1.57 (1.13–2.17) | 0.006 |
| Determinate *TP53* variant count only | Composite PFS | 93 | 76 | 1.44 (1.08–1.93) | 0.013 |
| Single variant with VAF >50% recoded as probably biallelic | OS | 93 | 60 | 1.49 (1.10–2.02) | 0.011 |
| Single variant with VAF >50% recoded as probably biallelic | Composite PFS | 93 | 76 | 1.37 (1.04–1.80) | 0.027 |

All models adjust for age, sex, *TP53* VAF, AML/MDS and complex karyotype. The analyses restricted to patients with a determinate *TP53* variant count additionally include the *TP53* grouping variable, defined first by variant count alone and then after recoding single-variant patients with a *TP53* variant allele frequency above 50% as probably biallelic; 21 of 37 single-variant patients (56.8%) met the latter criterion. Allogeneic HSCT was recorded without transplantation dates and was therefore not modeled as a time-dependent exposure; the first analysis addresses the resulting potential for immortal-time bias. VAF, variant allele frequency.

**Supplementary Table S8. Comparison of patients with and without C-reactive protein and ferritin measurement.**

| **Characteristic** | **CRP measured (n=56)** | **CRP not measured (n=58)** | **P** | **Ferritin measured (n=84)** | **Ferritin not measured (n=30)** | **P** |
| --- | --- | --- | --- | --- | --- | --- |
| Age, years | 64.5 (59.0–69.2) | 65.0 (58.2–73.0) | 0.348 | 65.0 (57.8–71.0) | 64.5 (60.2–70.0) | 0.977 |
| Male sex | 33 (58.9%) | 28 (48.3%) | 0.267 | 45 (53.6%) | 16 (53.3%) | 1.000 |
| AML | 30 (53.6%) | 24 (41.4%) | 0.260 | 36 (42.9%) | 18 (60.0%) | 0.137 |
| Complex karyotype | 43 (76.8%) | 39 (67.2%) | 0.301 | 56 (66.7%) | 26 (86.7%) | 0.057 |
| *TP53* variants ≥2 | 32 (57.1%) | 24 (41.4%) | 0.134 | 41 (48.8%) | 15 (50.0%) | 1.000 |
| *TP53* VAF, % | 64.0 (33.6–86.4) | 43.5 (13.9–74.8) | 0.024 | 42.8 (19.7–72.7) | 73.4 (56.2–88.9) | <0.001 |
| Marrow blasts, % | 21.2 (5.9–48.8) | 12.4 (3.5–35.8) | 0.063 | 13.0 (3.9–42.6) | 28.0 (10.8–44.2) | 0.103 |
| WBC, ×10⁹/L | 3.8 (2.3–12.4) | 3.0 (1.8–5.5) | 0.061 | 3.1 (2.0–6.8) | 3.4 (2.2–9.0) | 0.719 |
| LDH, U/L | 294 (187–492) | 265 (210–393) | 0.675 | 267 (194–388) | 341 (222–468) | 0.093 |
| Albumin, g/L | 34.7 (31.3–40.2) | 39.8 (36.3–42.0) | 0.002 | 38.1 (35.4–41.7) | 33.9 (30.9–39.8) | 0.025 |
| LAR, U/g | 8.5 (5.3–14.3) | 6.7 (5.2–9.8) | 0.189 | 6.6 (5.1–10.3) | 9.9 (6.0–13.6) | 0.029 |
| Supportive care only | 9 (16.1%) | 9 (15.5%) | 1.000 | 14 (16.7%) | 4 (13.3%) | 0.777 |
| Deaths | 39 (69.6%) | 35 (60.3%) | — | 54 (64.3%) | 20 (66.7%) | — |
| Adjusted HR for OS, measured vs not | 1.15 (0.72–1.85) | — | 0.554 | 0.85 (0.49–1.48) | — | 0.561 |
| Adjusted HR for composite PFS, measured vs not | 1.02 (0.67–1.55) | — | 0.931 | 0.80 (0.48–1.34) | — | 0.398 |

Values are median (IQR) or n (%). Continuous variables were compared with the Mann–Whitney test and categorical variables with the Fisher exact test. The final two rows give the hazard ratio for measurement status entered as a covariate in a Cox model adjusted for age, sex, *TP53* VAF, AML/MDS and complex karyotype. CRP and ferritin were measured at the discretion of the treating team and not by protocol. CRP, C-reactive protein; VAF, variant allele frequency; LAR, LDH-to-albumin ratio; OS, overall survival; PFS, progression-free survival.

**Supplementary Table S9. Disease-subgroup, stratified and interaction analyses for continuous LAR.**

| **Endpoint** | **Population or model** | **n** | **Events** | **Estimate per LAR doubling (95% CI)** | **P** |
| --- | --- | --- | --- | --- | --- |
| Overall survival | AML | 54 | 37 | HR 1.52 (1.06–2.17) | 0.022 |
| Overall survival | MDS | 60 | 37 | HR 1.93 (1.24–3.00) | 0.004 |
| Overall survival | Stratified by diagnosis | 114 | 74 | HR 1.62 (1.23–2.13) | <0.001 |
| Overall survival | Pooled (clinical model) | 114 | 74 | HR 1.57 (1.20–2.05) | <0.001 |
| Composite PFS | AML | 54 | 45 | HR 1.33 (0.97–1.81) | 0.072 |
| Composite PFS | MDS | 60 | 48 | HR 1.82 (1.22–2.74) | 0.004 |
| Composite PFS | Stratified by diagnosis | 114 | 93 | HR 1.46 (1.15–1.87) | 0.002 |
| Composite PFS | Pooled (clinical model) | 114 | 93 | HR 1.44 (1.14–1.84) | 0.003 |
| Progression, cause-specific | AML | 54 | 35 | HR 1.16 (0.79–1.70) | 0.455 |
| Progression, cause-specific | MDS | 60 | 41 | HR 2.01 (1.30–3.09) | 0.002 |
| Progression, cause-specific | Stratified by diagnosis | 114 | 76 | HR 1.43 (1.08–1.91) | 0.013 |
| Progression, cause-specific | Pooled (clinical model) | 114 | 76 | HR 1.39 (1.05–1.83) | 0.021 |
| Progression, Fine–Gray | AML | 54 | 35 | sHR 0.79 (0.57–1.09) | 0.156 |
| Progression, Fine–Gray | MDS | 60 | 41 | sHR 1.73 (1.14–2.61) | 0.009 |
| Progression, Fine–Gray | Pooled (clinical model) | 114 | 76 | sHR 1.02 (0.80–1.31) | 0.855 |

Within-diagnosis and stratified models adjust for age, sex, *TP53* VAF and complex karyotype; the pooled clinical model additionally includes the AML/MDS indicator. Stratified models allow a separate baseline hazard for AML and MDS; no stratified estimate is reported for the Fine–Gray model, for which stratification of the baseline subdistribution hazard was not fitted. Exploratory likelihood-ratio tests for a LAR-by-diagnosis interaction were not significant (P=0.465 for overall survival and P=0.393 for composite PFS); the study was not powered to detect an interaction and these subgroup estimates are descriptive. Median LAR was 8.97 in AML and 6.39 in MDS. HR, hazard ratio; sHR, subdistribution hazard ratio.
